# Supplementary material for: Probiotic Effects of a Marine Purple Non-Sulfur Bacterium, Rhodovulum sulfidophilum KKMI01, on Kuruma Shrimp (Marsupenaeus japonicus)
Source: Microorganisms. 2022 Jan 22;10(2):244. doi: 10.3390/microorganisms10020244 (PMC8876596; doi:10.3390/microorganisms10020244)
Supplement: Supplementary file 1 [file microorganisms-10-00244-s001.zip › Supple Figure S1.pptx]

## Slide 1
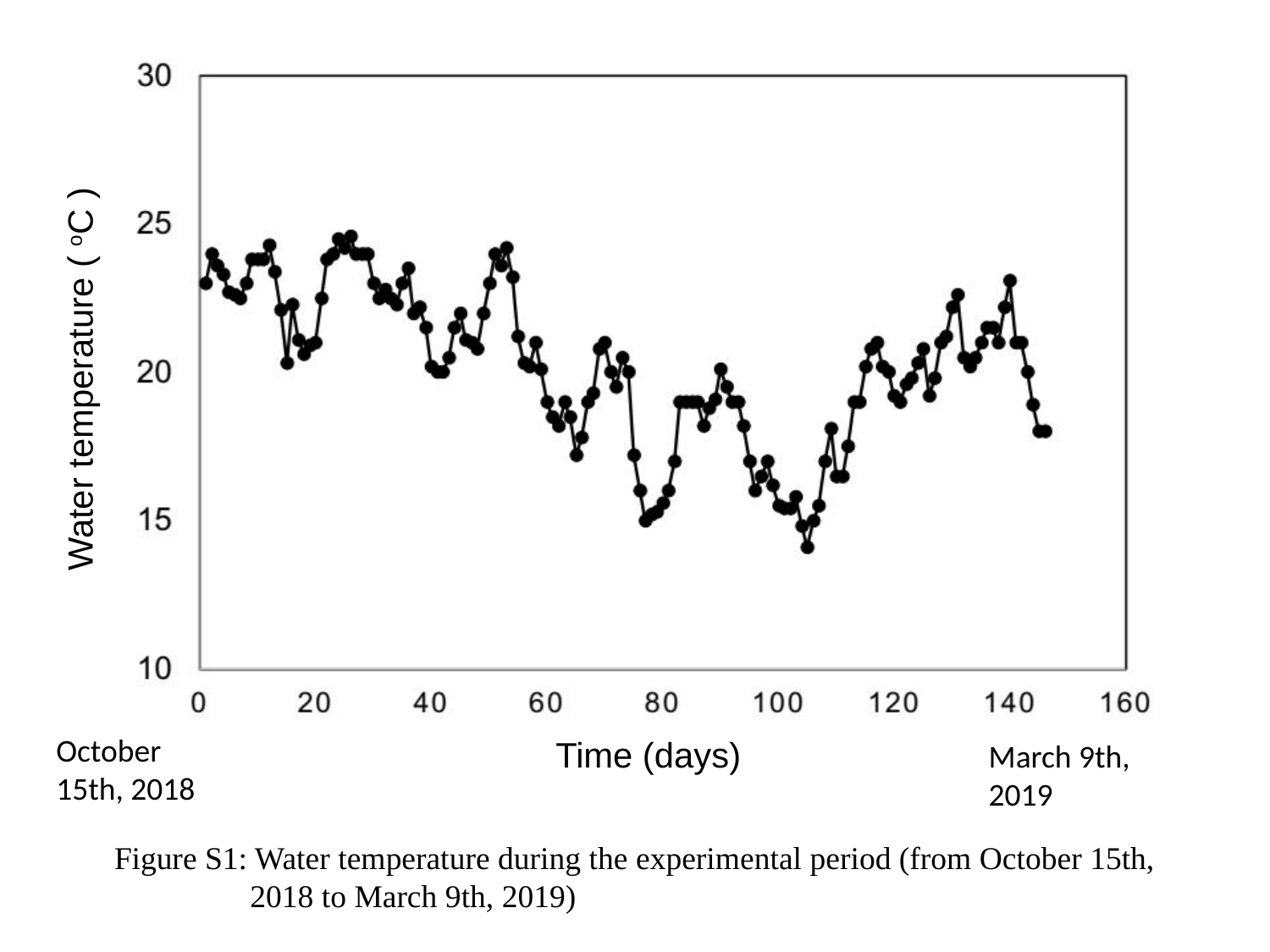

Water temperature ( oC )
October 15th, 2018
Time (days)
March 9th, 2019
Figure S1: Water temperature during the experimental period (from October 15th, 2018 to March 9th, 2019)
