# Supplementary material for: Probiotic Effects of a Marine Purple Non-Sulfur Bacterium, Rhodovulum sulfidophilum KKMI01, on Kuruma Shrimp (Marsupenaeus japonicus)
Source: Microorganisms. 2022 Jan 22;10(2):244. doi: 10.3390/microorganisms10020244 (PMC8876596; doi:10.3390/microorganisms10020244)
Supplement: Supplementary file 1 [file microorganisms-10-00244-s001.zip › Supple Figure S2.pptx]

## Slide 1
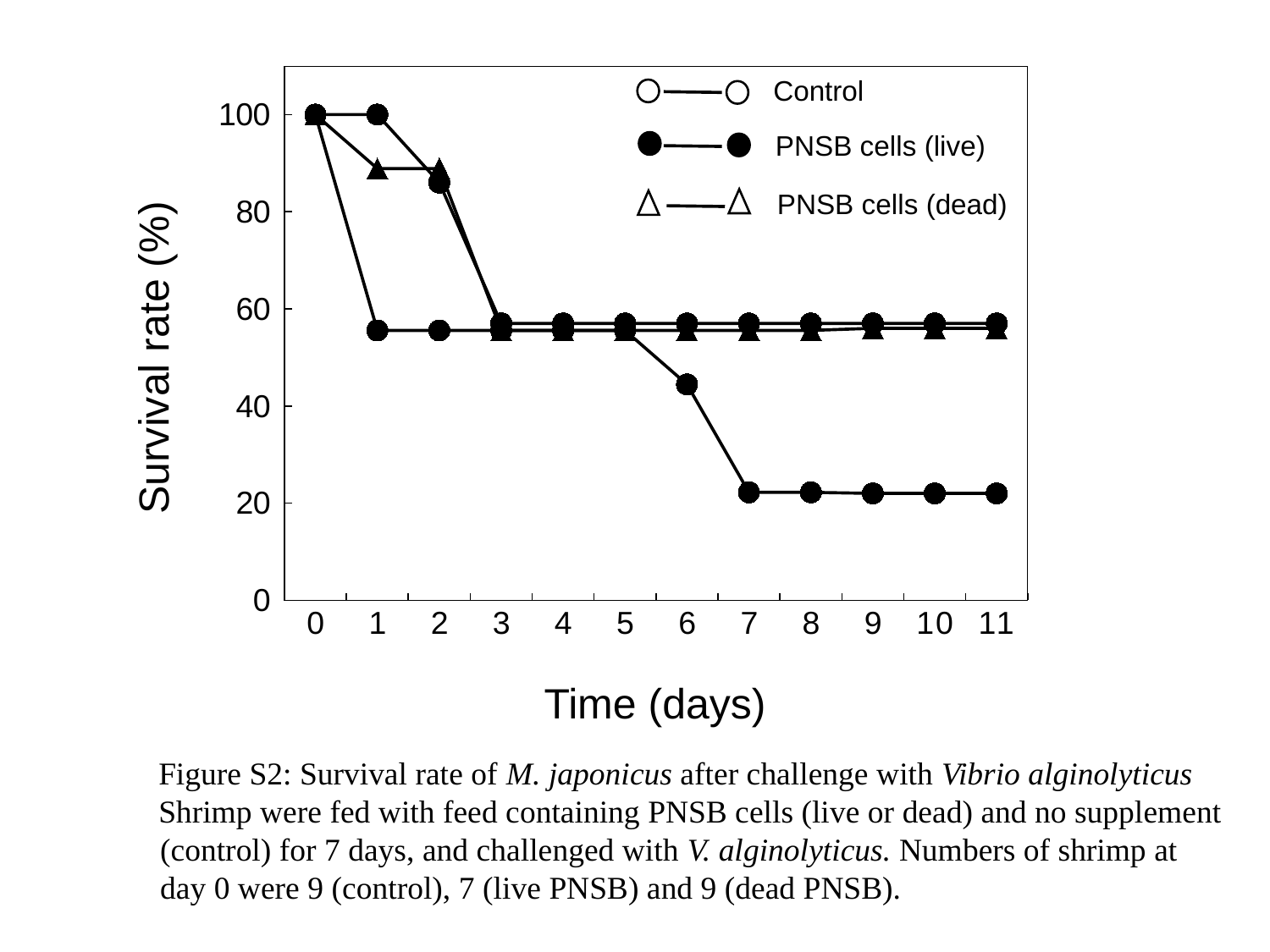

### Chart
| Category | Control | KKMI01(生) | KKMI01(死) |
|---|---|---|---|
| 0 | 100.0 | 100.0 | 100.0 |
| 1 | 55.55555555555556 | 100.0 | 88.88888888888889 |
| 2 | 55.55555555555556 | 86.0 | 88.88888888888889 |
| 3 | 55.55555555555556 | 57.0 | 55.55555555555556 |
| 4 | 55.55555555555556 | 57.0 | 55.55555555555556 |
| 5 | 55.55555555555556 | 57.0 | 55.55555555555556 |
| 6 | 44.44444444444444 | 57.0 | 55.55555555555556 |
| 7 | 22.22222222222222 | 57.0 | 55.55555555555556 |
| 8 | 22.22222222222222 | 57.0 | 55.55555555555556 |
| 9 | 22.0 | 57.0 | 56.0 |
| 10 | 22.0 | 57.0 | 56.0 |
| 11 | 22.0 | 57.0 | 56.0 |Control
PNSB cells (live)
PNSB cells (dead)
Survival rate (%)
Time (days)
Figure S2: Survival rate of M. japonicus after challenge with Vibrio alginolyticus
Shrimp were fed with feed containing PNSB cells (live or dead) and no supplement (control) for 7 days, and challenged with V. alginolyticus. Numbers of shrimp at day 0 were 9 (control), 7 (live PNSB) and 9 (dead PNSB).

## Slide 2
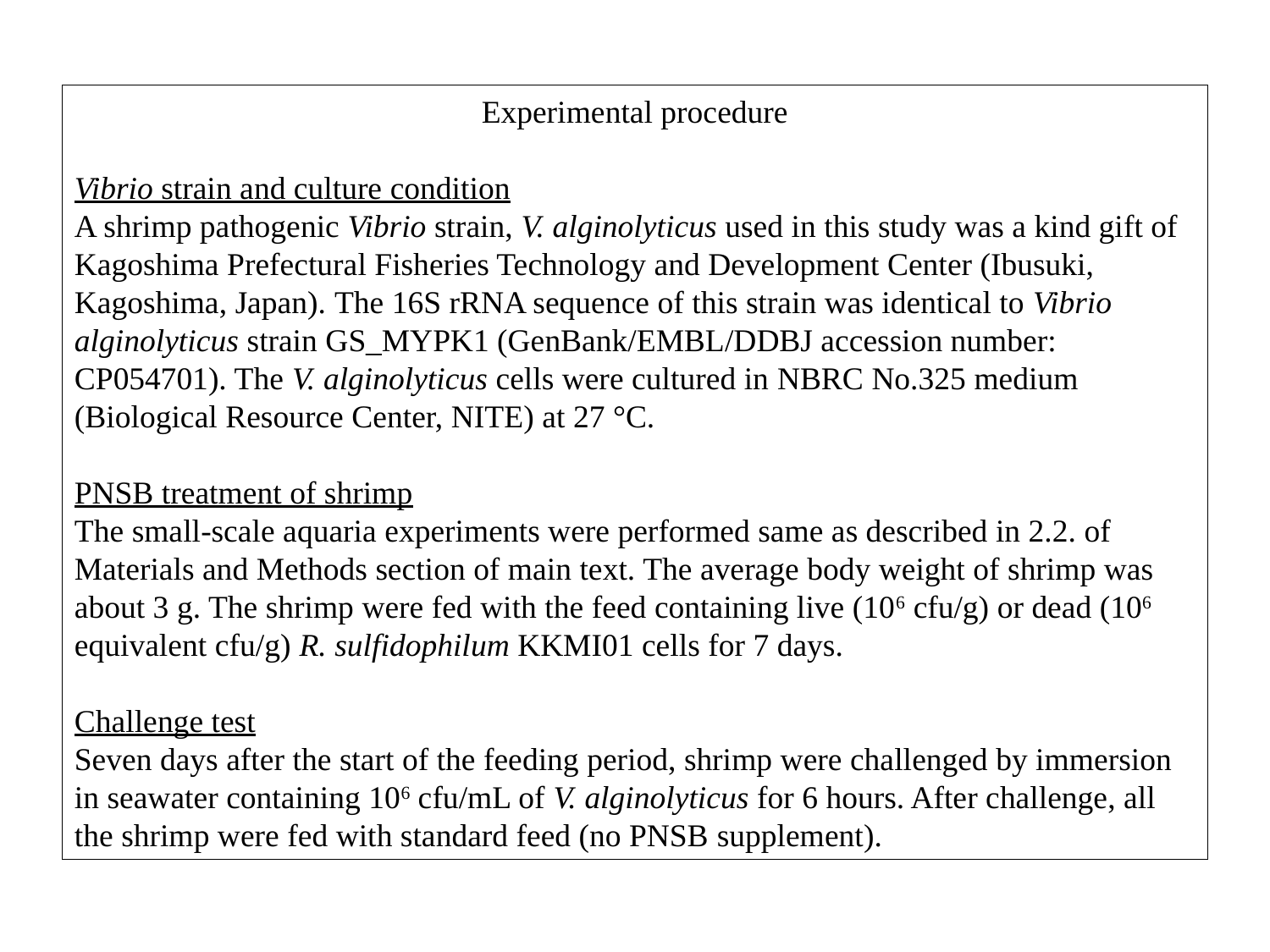

Experimental procedure
Vibrio strain and culture condition
A shrimp pathogenic Vibrio strain, V. alginolyticus used in this study was a kind gift of Kagoshima Prefectural Fisheries Technology and Development Center (Ibusuki, Kagoshima, Japan). The 16S rRNA sequence of this strain was identical to Vibrio alginolyticus strain GS_MYPK1 (GenBank/EMBL/DDBJ accession number: CP054701). The V. alginolyticus cells were cultured in NBRC No.325 medium (Biological Resource Center, NITE) at 27 °C.
PNSB treatment of shrimp
The small-scale aquaria experiments were performed same as described in 2.2. of Materials and Methods section of main text. The average body weight of shrimp was about 3 g. The shrimp were fed with the feed containing live (106 cfu/g) or dead (106 equivalent cfu/g) R. sulfidophilum KKMI01 cells for 7 days.
Challenge test
Seven days after the start of the feeding period, shrimp were challenged by immersion in seawater containing 106 cfu/mL of V. alginolyticus for 6 hours. After challenge, all the shrimp were fed with standard feed (no PNSB supplement).
