# Supplementary material for: Probiotic Effects of a Marine Purple Non-Sulfur Bacterium, Rhodovulum sulfidophilum KKMI01, on Kuruma Shrimp (Marsupenaeus japonicus)
Source: Microorganisms. 2022 Jan 22;10(2):244. doi: 10.3390/microorganisms10020244 (PMC8876596; doi:10.3390/microorganisms10020244)
Supplement: Supplementary file 1 [file microorganisms-10-00244-s001.zip › Supple Figure S3.pptx]

## Slide 1
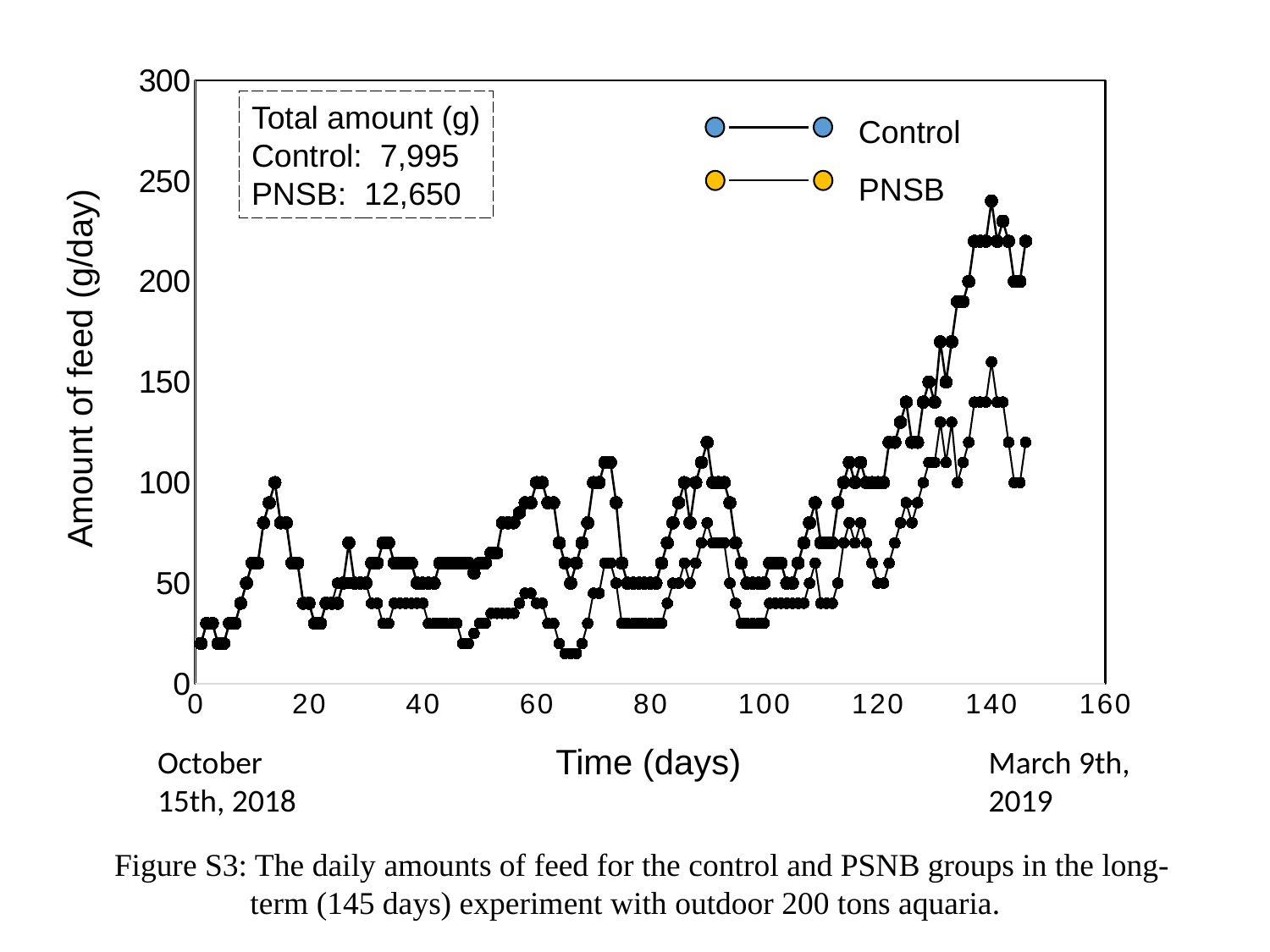

### Chart
| Category | 対照区（g） | 投与区（g） |
|---|---|---|Control
PNSB
Total amount (g)
Control: 7,995
PNSB: 12,650
Amount of feed (g/day)
Time (days)
March 9th, 2019
October 15th, 2018
Figure S3: The daily amounts of feed for the control and PSNB groups in the long-term (145 days) experiment with outdoor 200 tons aquaria.
