# Supplementary material for: Probiotic Effects of a Marine Purple Non-Sulfur Bacterium, Rhodovulum sulfidophilum KKMI01, on Kuruma Shrimp (Marsupenaeus japonicus)
Source: Microorganisms. 2022 Jan 22;10(2):244. doi: 10.3390/microorganisms10020244 (PMC8876596; doi:10.3390/microorganisms10020244)
Supplement: Supplementary file 1 [file microorganisms-10-00244-s001.zip › Text S1 Primer list.pdf]

Supplemental Text S1. List of primers used for qRT-PCR

Cuticular protein DD1 (AB194409)
DD1_qF 5’-GGTTCTCGCCTTACCCCTTC-3’
DD1_qR 5’-AGGATTTCCGCTAGCATCCG-3’

Cuticular protein DD5 (AB049147)
DD5_qF 5’-CAGGGTCACAGGGAGTTCTG-3’
DD5_qR 5’-CTTCTCGGGCACTGGTATCG-3’

Cuticular protein DD9A (AB031223)
DD9A-qF 5’-ATGAGAATGGCTTCGTCCCC-3’
DD9A-qR 5’-CGGCGATTTCAAGGAGTTCG-3’

Cuticular protein DD9B (AB031224)
DD9B-qF 5’-AGAAGTCAGATTCACCGCCG-3’
DD9B-qR 5’-GTTGGTCGGCGATTTCAAGG-3’

Crustocalcin-b (AB114445)
CrustocalcinB-qF 5’-GCCGAGGGAAAGTCGGAAGC-3’
CrustocalcinB-qR 5’-GGGCTTTCTCGACATCAGCA-3’

Ca^{2+}-ATPase (CI999271)
Ca-ATPase-qF 5’-TCCAGCTCTTGTGGGTCAAC-3’
Ca-ATPase-qR 5’-GCGAGGAGGTTTGTCCATGA-3’

Crustacyanin_A (FJ498897)
Crustacyanin_A-qF 5’-TACACTCGCTGCATCCACTC-3’
Crustacyanin_A-qR 5’-GTACTCGTTGTTGGGGCTGA-3’

Myosin heavy chain type b (MYHb) (AB613206)
MYHb-qF 5’-TCAGCATGGATCCCTCTCCA-3’
MYHb-qR 5’-GGAGTTCTTGGCCTCGTTCA-3’

Prophenoloxidase (AB065371)
proPO-F 5’-TCCAAGTGCCAGAACGAAATG-3’

proPO-R 5’-CGATGAGACGCGAGGAAGA-3’

SOD-2 (AB908996)

SOD2qF 5’-AGTGACCCCTTAGTGAGCCT-3’

SOD2qR 5’-GGTCTTCAAGCTGTCCTCGT-3’

Heat shock protein 70 (HSP70) (DQ663761)

hsp70-qF 5’-ATCACTCGTGCTCGCTTTGA-3’

hsp70-qR 5’-ATCTTGGGGATACGGGTGGA-3’

Diphthamide biosynthesis protein 7 (Dph7) (MG566082)

Dph7-qF 5’-AAACGACTTGGGCGTCTCTACC-3’

Dph7-qR 5’-GGCAAGCATTGGTGTTCCTGATA-3’

Serine proteinase inhibitor (Serpin) (AB505891)

Serpin-qF 5’-TTTGGTCGGCCTTCATCCTC-3’

Serpin-qR 5’-CCCGCCAGATCTTGAAGGTT-3’

Toll-like receptor 1 (AB333779)

TLR1-F 5’-TCGCCAGTACAGTGATCAGCAT-3’

TLR1-R 5’-CATTCACCACAGCCCACAAG-3’

Toll-like receptor 2 (AB385869)

TLR2-F 5’-CCTTCTCCTGTTTGCTGTCCTT-3’

TLR2-R 5’-GCATCTAATTCGTCCTCTGTTATGG-3’

Dorsal (KX424930)

Dorsal-qF 5’-AGACTGGGTTTTCTCATCGTAATC-3’

Dorsal-qR 5’-TAAATGGGATCTGACACTTGTGG-3’

Antilipopolysaccharide factor (ALF) (KX424931)

AntLPS_KX424931_qF 5’-GATTGTGGCAGACCGGAGAA-3’

AntLPS_KX424931_qR 5’-CATGCGACCCCTGAAGTACA-3’

Antilipopolysaccharide factor D (ALF-D) (MN416688)

AntLPS_D_qF 5’-CAACTTTCGTAGGCGCTGTG-3’

AntLPS_D_qR 5’-GTTCCGGTCCTCCATAAGCC-3’

Crustin-like peptide (AB121740)

Crustin-F 5’-GGTCGAGCGACTGCAGGTA-3’

Crustin-R 5’-GGCAGTCCAGTGGCTTGGTA-3’

IMD (Immune deficiency)(AB478859)

IMDqF 5’-TGGAATGAAGCCCTGTTCCC-3’

IMDqR 5’-TGCCAAGGTGTTCGCTTACT-3’
